# Asymptomatic diabetes: screening by routine imaging beneficial?

**DOI:** 10.1007/s12471-014-0643-8

**Published:** 2014-12-19

**Authors:** Ernst E. van der Wall

**Affiliations:** Holland Heart House/Netherlands Society of Cardiology, Moreelsepark 1, 3511 EP Utrecht, The Netherlands

According to the 2013 ESC Guidelines on diabetes, pre-diabetes, and cardiovascular diseases, developed in collaboration with the European Association for the Study of Diabetes (EASD), approximately 360 million people worldwide had diabetes in 2011, of whom > 90 % with type 2 diabetes [1]. This number will increase to 552 million by the year 2030. Another 300 million individuals showed features indicating future risk of developing type 2 diabetes, including fasting hyperglycaemia, impaired glucose tolerance, gestational diabetes and euglycaemic insulin resistance. The majority of new cases of type 2 diabetes occur in the context of Westernised lifestyles, high-fat diets and decreased exercise, leading to increasing levels of obesity, insulin resistance and, ultimately, beta-cell failure and type 2 diabetes [2]. The Middle East, the Asia–Pacific rim and parts of both North and South America have experienced massive increases in the prevalence of diabetes over the past 20 years, changes mirrored in European populations over the same period. For Europe, the International Diabetes Federation’s global estimates for 2011 suggested that 52 million Europeans aged 20–79 years have diabetes and that this number will increase to over 64 million by the year 2030. The healthcare expenditure for diabetes in Europe was about 75 billion € in 2011 and is projected to increase to 90 billion € by the year 2030. Awareness of specific issues associated with gender and race and, particularly, the effects of diabetes in women are becoming of increasing importance.

For the Netherlands, in 2011 a total of 830,000 individuals (415,900 male, 418,200 female) were known with diabetes mellitus, of whom 90 % with type 2 diabetes. Prevalence was highest in the age group between 75 and 85 years and already 600,000 individuals between 30 and 70 years were known to have diabetes [3].

Patients with diabetes have a high risk of adverse cardiac events [4, 5]. A total of 281 million men and 317 million women worldwide with diabetes died in 2011, most from cardiovascular disease in particular coronary artery disease (CAD). CAD is often silent in diabetes patients and up to 60 % of myocardial infarctions may be asymptomatic, diagnosed only by ECG stress testing, myocardial scintigraphy or stress echocardiography. Silent myocardial ischaemia affects 20–35 % of diabetes patients who have additional risk factors, and 35–70 % of patients with silent ischaemia have significant coronary stenosis at coronary arteriography.

In asymptomatic patients, routine screening for CAD is controversial [6–8]. It is not recommended by the American Diabetes Association (ADA), since it does not improve outcomes as long as cardiovascular risk factors are treated. This position is, however, under serious debate and the characteristics of the patients who should be screened for CAD need to be better defined. To that purpose, several imaging strategies have proposed to screen for CAD in asymptomatic diabetic patients [9–14].

To further expand the value of imaging strategies in asymptomatic diabetes, the FACTOR-64 trial was performed and presented at the November 2014 meeting of the American Heart Association (AHA) and simultaneously published in the Journal of the American Medical Association (JAMA) [15]. The FACTOR-64 trial is entitled: *Screening for Asymptomatic Obstructive Coronary Artery Disease Among High-Risk Diabetic Patients Using CT Angiography*. This randomised, blinded parallel trial evaluated the efficacy of routine screening for CAD in patients with type 1 or 2 diabetes and no overt symptoms of CAD. The hypothesis was that CAD screening with coronary computed tomography angiography (CCTA) in patients with diabetes mellitus and no known CAD would reduce cardiovascular outcomes compared with routine management, i.e. standard national guidelines–based optimal diabetes care. It was assumed that routine CCTA-guided CAD screening and directed therapy would be superior to routine management in cardiovascular risk reduction in patients with diabetes of at least 3–5 years’ duration. Coronary arteriography and coronary artery calcium (CAC) levels were performed on a Toshiba Aquillon 64 CT scanner. Scan results were classified as severe stenosis (≥ 70 % stenosis in at least one major proximal or large coronary artery; recommendation to pursue coronary angiography), moderate stenosis (50–69 % stenosis or CAC > 100; recommendation to pursue stress testing), mild stenosis (10–49 % stenosis or CAC > 10–100; no further imaging recommended) or normal (< 10 % stenosis and CAC ≤ 10; no further imaging recommended).

In the FACTOR 64-trial, a total of 900 patients were randomised: 452 to CCTA and 448 patients to routine management. The patient population consisted of men ≥ 50 years old with at least a 3-year history of diabetes or ≥ 40 years old with at least a 5-year history of diabetes (52 %), and women ≥ 55 years old with at least a 3-year history of diabetes or ≥ 45 years old with at least a 5-year history of diabetes, all patients on antidiabetic medication for at least 1 year. Baseline characteristics were fairly similar between the two arms. Mean duration of diabetes was 13 years, and 20 % of the patients were on insulin only, with another 23 % on both insulin and noninsulin agents. The primary endpoint was a composite of all-cause mortality, nonfatal myocardial infarction, and hospitalisation for unstable angina.

In the CCTA arm, the median CAC score was 55, with 41 % having Agatston scores ≥ 100. About 21 % had moderate or severe stenosis on CCTA. An aggressive medical regimen was adopted in 70 % of subjects in the CCTA arm. The primary endpoint was similar between the CCTA and routine management arms (6.2 vs. 7.6 %). All-cause mortality (3.5 vs. 4.3 %), nonfatal myocardial infarction (1.5 vs. 1.8 %), cardiovascular death (1.5 vs. 1.8 %), CAD death (1.1 vs. 0.4 %) and stroke/carotid revascularisation procedure (1.8 vs. 2.0 %) were all similar between the two arms; hospitalisation for congestive heart failure was lower in the CCTA arm (0.7 vs. 2.2 %, *p* = 0.04).

To summarise, the results of the FACTOR-64 trial indicate that routine screening for CAD in asymptomatic patients with diabetes using CCTA was not beneficial in reducing cardiovascular events compared with routine management. To put it short: these data do not support routine CCTA screening in this population. The findings rather suggest that focused prevention with optimal guideline-directed medical therapy in asymptomatic patients with diabetes is far more important than using a routine cardiac imaging strategy. This reinforces the importance of aggressive risk factor and lifestyle modification in patients with diabetes mellitus.
